# Quantitative flow ratio-based outcomes in patients undergoing transcatheter aortic valve implantation quaestio study

**DOI:** 10.3389/fcvm.2023.1188644

**Published:** 2023-08-30

**Authors:** Pierluigi Demola, Iginio Colaiori, Davide Bosi, Sergio Musto D’Amore, Marco Vitolo, Giorgio Benatti, Luigi Vignali, Iacopo Tadonio, Davide Gabbieri, Luciano Losi, Paolo Magnavacchi, Fabio Alfredo Sgura, Giuseppe Boriani, Vincenzo Guiducci

**Affiliations:** ^1^ Cardiology Unit, Azienda USL—IRCCS di Reggio Emilia, Reggio Emilia, Italy; ^2^ Cardiology Division, Department of Biomedical, Metabolic and Neural Sciences, University of Modena and Reggio Emilia, Policlinico di Modena, Modena, Italy; ^3^ Clinical and Experimental Medicine PhD Program, University of Modena and Reggio Emilia, Modena, Italy; ^4^ Cardiology Department, Azienda Ospedaliero-Universitaria of Parma, Parma, Italy; ^5^ Cardiac Surgery Division, Hesperia Hospital, Modena, Italy; ^6^ U.O. Cardiologia, Ospedale “Guglielmo da Saliceto”, Piacenza, Italy; ^7^ Cardiology Division, Baggiovara Hospital, Modena, Italy

**Keywords:** aortic stenosis, coronary artery disease, coronary physiology, fractional flow reserve, quantitative flow ratio (QFR)

## Abstract

**Background:**

Coronary artery disease (CAD) is common in patients with aortic valve stenosis (AS) ranging from 60% to 80%. The clinical and prognostic role of coronary artery lesions in patients undergoing Transcatheter Aortic Valve Implantation (TAVI) remains unclear. The aim of the present observational study was to estimate long-term clinical outcomes by Quantitative Flow Ratio (QFR) characterization of CAD in a well-represented cohort of patients affected by severe AS treated by TAVI.

**Methods:**

A total of 439 invasive coronary angiographies of patients deemed eligible for TAVI by local Heart Teams with symptomatic severe AS were retrospectively screened for QFR analysis. The primary endpoint of the study was all-cause mortality. The secondary endpoint was a composite of cardiovascular mortality, stroke/transient ischemic attack (TIA), acute myocardial infarction (AMI), and any hospitalization after TAVI.

**Results:**

After exclusion of patients with no follow-up data, coronary angiography not feasible for QFR analysis and previous surgical myocardial revascularization (CABG) 48/239 (20.1%) patients had a QFR value lower or equal to 0.80 (QFR + value), while the remaining 191/239 (79.9%) did not present any vessel with a QFR positive value. In the adjusted Cox regression analysis, patients with positive QFR were independently associated with an increased risk of all-casual mortality (Model 1, HR 3.47, 95% CI, 2.35−5.12; Model 2, HR 5.01, 95% CI, 3.17−7.90). In the adjusted covariate analysis, QFR+ involving LAD (37/48, 77,1%) was associated with the higher risk of the composite outcome compared to patients without any positive value of QFR or non-LAD QFR positive value (11/48, 22.9%).

**Conclusions:**

Pre-TAVI QFR analysis can be used for a safe, simple, wireless functional assessment of CAD. QFR permits to identify patients at high risk of cardiovascular mortality or MACE, and it could be considered by local Heart Teams.

## Introduction

Coronary artery disease (CAD) is common in patients with aortic valve stenosis (AS) ranging from 60% to 80% (1–3). AS and CAD inherently share several cardiovascular risk factors (4) and the patients baseline characteristics can easily overlap. Angina *per se*, and other equivalent symptoms in the setting of AS have low specificity and poor positive predictive value discerning valvulopathy from CAD.

Meanwhile, Transcatheter Aortic Valve Implantation (TAVI) is rapidly becoming the first-line treatment of severe AS, competing with the traditional Surgical Aortic Valve Replacement (SAVR) strategy even in low-risk patients (5, 6). According to European guidelines, TAVI is the recommended therapeutic choice for severe symptomatic AS in patients aged 75 years or older and can be considered in patients from 65 to 75 years based on patient/anatomy characteristics and shared decision-making by the local Heart Team (7). American Guidelines recommend TAVI even in patients with a lower threshold, from 65 years old (8).

The clinical and prognostic role of coronary artery lesions in patients undergoing TAVI remains controverse and evidence are limited and still debated (3, 9–14). The bottom line is that CAD has been associated with a shorter survival rate in patients undergoing TAVI (15, 16). Percutaneous coronary intervention (PCI) and subsequent dual antiplatelet therapy (DAPT) are burdened with several complications and an increased bleeding risk, especially in elderly patients treated with TAVI (17).

ICA is pivotal in the assessment of CAD of TAVI candidates (7, 18).

Addition of physiological guidance for CAD can optimize patient outcomes (19, 20). Functional assessment of CAD refers to the physiological evaluation of the hemodynamic impact of coronary artery stenosis based on its effect on blood flow through the vessel (21). It implies the physiological study of the coronary arteries by hyperemia or non-hyperemic methodologies. Since the mere angiographic percentage of stenosis alone cannot accurately reflect the hemodynamic significance of the lesion, functional assessment techniques such as FFR, non-hyperemic indices, and lately QFR, that can provide useful information for clinical decision-making. The functional assessment of CAD demonstrated superiority over the evaluation of angiography for guiding PCI in patients with intermediate stenosis, but patients with severe valvulopathies were excluded from the main trials (19, 22, 23), so data are limited in this scenario. The optimal management of patients with severe AS and concomitant CAD in patients undergoing TAVI is controversial and adenosine-based assessment methods has shown conflicting results (24–27). Secondly, there is an increasing emphasis on the prediction of long-term outcomes in patients with significant vessel stenoses and, moreover, on the challenging re-access of coronary ostia after the implant of a Transcatheter Heart Valve (THV) and on the optimal procedural timing. Stratification of this heterogeneous behavior of CAD in AS setting has led to the quest of more sophisticated approaches, such as risk scores and non-invasive functional assessment of CAD (11, 28, 29).

Quantitative Flow Ratio (QFR) is a fast computational modality of FFR based on 3-dimensional quantitative coronary angiography (3D-Quantitative Coronary Angiography) and contrast flow velocity during ICA.

Recent advances in coronary physiology have shown that QFR has a good diagnostic performance compared to Fractional Flow Reserve (FFR) and it is superior compared to angiography alone and safer in assessing the functional relevance of coronary lesions (30), even in TAVI patients (16, 31). The optimal approach was validated in the FAVOR (Functional Assessment by Various Flow Reconstructions) study saga, proving the reliability of QFR (30) and its superiority to angiographic assessment for evaluation of intermediary coronary artery stenosis, using FFR as a reference standard (32).

The present QUAESTIO study, QUAntitative flow ratio-based outcomES in paTIents undergoing trans catheter aOrtic Valve Implantation**,** is the first QFR-based analysis study aimed at estimating long-term clinical outcomes by QFR characterization of CAD in a well-represented cohort of consecutive patients with severe AS treated by TAVI.

## Methods

The present study was a retrospective and observational study. The QUAESTIO study was done after approval by the Italian Ethic Committee of AVEN (Area Vasta Emilia Nord) as multicenter observational retrospective study. A total of 439 ICAs of patients deemed eligible for TAVI between July 2010 and December 2019 by local Heart Teams with symptomatic severe AS were retrospectively screened. Overall, 239/439 (54.4%) presented complete follow-up data, analyzable QFR according to the criteria shown in Supplementary Figure S1 and had not previously been treated by surgical myocardial revascularization (CABG), had no hemodynamic instability at the time of ICA, and patients without acute coronary syndrome.

All ICAs were performed in five cardiology Italian centers: Azienda USL-IRCCS Reggio Emilia, Ospedale Maggiore di Parma, Ospedale di Baggiovara, Policlinico di Modena and Hesperia Hospital in Modena, all located in Emilia Romagna, a northern Italian region. The TAVI was staged in two different hub centers: Parma (University Hospital of Parma) and Modena (Hesperia Hospital) with cardiac surgery facilities, following guidelines indications (7).

### Invasive coronary angiography and QFR analysis

Overall, 239/439 complete ICAs (54,4%) were finally analyzed by QFR.

Patient-specific exclusion criteria included: age less than 18 years, previous coronary artery bypass grafting, hemodynamic instability at the time of ICA and patients with acute coronary syndrome.

Coronary-specific exclusion criteria included: low images quality, excessive vessel overlaps or tortuosity, coronary aneurysms, aorto-ostial lesions, or absence of valid projections (all exclusion criteria are summarized Supplementary Figure S1).

ICAs percutaneous access was for most cases radial, more rarely femoral. After administration of low dose of intracoronary nitroglycerin (100−200 µg), angiographic views were acquired. All images were obtained at 15 frames per second and judged to be of good quality by the operator. We examined the investigated vessel for the availability of 2 orthogonal study projections (at least with 25-degree difference), with good contrast filling, without excessive overlap, shortcut of the investigated lesion or sliding of angiographic image.

Since no firm recommendations were available before 2021, the policy generally adopted by our centers was to not perform a routine revascularization of angiographic lesions, favoring treatment of aortic valvulopathy first also in line with the previous Guidelines (33, 34). This policy was adopted by centers primarily based on the final decision and angiographic assessment made by the physician at the time of coronary angiography; therefore, this strategy should be considered in light of an operator-dependent assessment of the coronary lumenogram.

The QFR assessment was performed retrospectively in all eligible vessels (right coronary artery, RCA; left anterior descendant artery, LAD; and left circumflex artery, LCx; with a minimum diameter of at least >2.0 mm) by two fully certified physicians (IC and DB, who obtained the Medis QAngio XA 3D analytical software solution certification before this study). Both were blinded to clinical outcomes and patients' characteristics and used the same dedicated QFR system (Medis Medical Imaging bv, The Netherlands). The QFR analysis was performed accurately following a standard step-by-step procedure collecting a total of 717 vessel QFR values (see the example and workflow chart in Supplementary Figure S1).

The vessels analyzed were defined as susceptible of ischemia if QFR value was ≤0.80, as validated in previous studies (32, 35). Patients in whom at least one of the three coronary vessels was labeled “functionally ischemic” were defined as having a positive QFR (QFR+); otherwise, they were defined as having a negative QFR (QFR-), as each coronary artery exceeded the QFR limit beyond 0.80.

The QFR+ group was further stratified into 2 subgroups according to the QFR positive value detected on left anterior descendant coronary artery (LAD), such as patients with physiologically significant ischemic LAD [baseline QFR <0.80 (involving LAD QFR+)], independently from other coronaries QFR value (non-LAD QFR+).

Finally, a retrospective comparison between the obtained angiographic results [both of percentage stenosis analysis using Quantitative Coronary Angiography (QCA) and proper vascular segment involved] and QFR assessment was also performed.

#### Endpoints and follow-up

The primary endpoint of the present study was all-cause mortality.

The secondary endpoint was a composite of all cause of death, cardiovascular mortality, stroke/transient ischemic attack (TIA), myocardial infarction (MI), and any hospitalization after TAVI.

**Figure 1:**
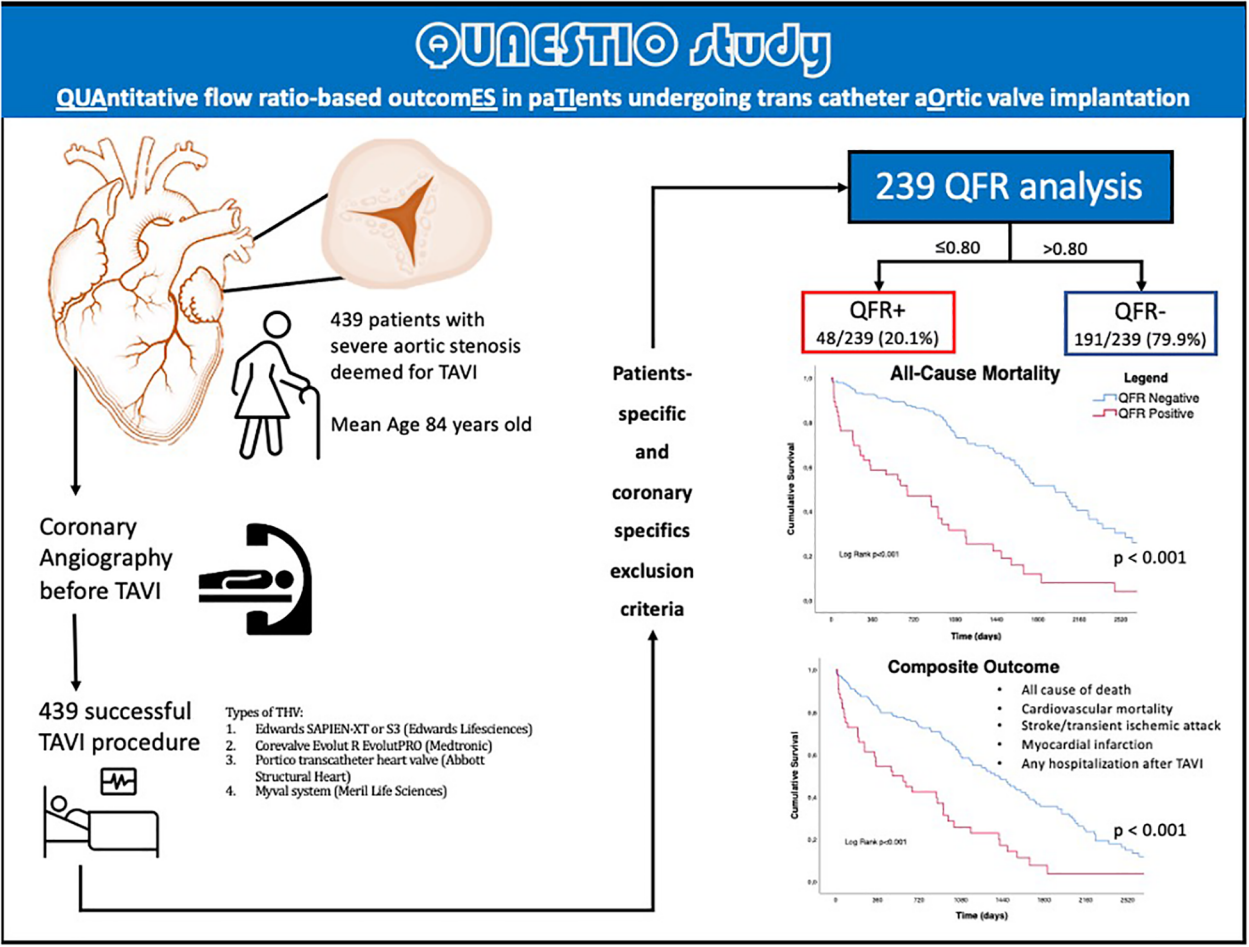
Central illustration. QFR, quantitative flow ratio; TAVI, transcatheter aortic valve implantation, TIA, transient ischemic attack; THV, transcatheter heart valve.

The median follow-up was 1005 days [IQR 582-2218]. A central illustration summarizes the workflow applied in this retrospective study, in Figure 1.

### TAVI procedure

TAVI procedures were performed in “Ospedale Maggiore” of Parma or in “Hesperia Hospital” of Modena by experienced interventional cardiologists and cardiac surgeons, either by the percutaneous transfemoral, and rarely by transapical approach or surgical subclavian access. The main implanted THVs were Edwards SAPIEN-XT or S3 (Edwards Lifesciences, Irvine, CA), Medtronic CoreValve, Evolut R or EvolutPro (Medtronic Inc., Minneapolis, MN), Portico transcatheter heart valve (Abbott Structural Heart, St Paul, MN, USA) and Myval system (Meril Life Sciences Pvt Ltd., Vapi, India). The choice of THV was based upon the anatomical features of the native valve morphology and only after a careful study of the vascular accesses, analyzed by computerized angiotomography.

#### Statistical analysis

Continuous variables were reported as median and interquartile range (IQR) or mean and standard deviation (SD). Non-parametric test (Mann-Whitney U) was used to perform among-group comparisons. Categorical variables were described as counts and percentages and among-group comparisons were made using a *χ*^2^ test or Fisher's exact test (if any expected cell count was less than five).

Plots of Kaplan–Meier curves for time to all-cause mortality and the composite outcome according to the QFR value (positive vs. negative) were performed. Survival distributions were compared using the log-rank test.

Unadjusted and adjusted Cox regression analyses were used to establish the relationship between QFR and the risk of outcomes. We built two different multivariable models introducing into the models a series of variables known to be predictors of adverse outcomes in TAVI patients: Model 1 was adjusted for age and sex; Model 2 was adjusted for age, sex, hypertension, diabetes, coronary artery disease, CKD, previous stroke, active malignancy, and atrial fibrillation. Results were expressed as hazard ratio (HR) and 95% confidence interval (CI).

A two-sided *p*-value <0.05 was considered statistically significant. All analyses were performed using SPSS statistical software (version 26.0, Statistical Package for the Social Sciences, SPSS, IBM, Chicago, Illinois).

## Results

### Baseline clinical and anamnestic characteristics

A total of 239 patients were included [median age 84 (IQR 81-86), 57.3% females]. Table 1 summarizes the baseline clinical characteristics of this study populations. Overall, 48/239 (20.1%) patients presented a QFR value lower or equal to 0.80 at the retrospective off-line analysis (QFR+ group), while the remaining 191/239 (79.9%) did not present any vessel with a QFR positive value (QFR- group).

**Table 1:** Baseline clinical characteristics according to QFR.

	Negative QFR (*n* = 191, 79.9%)	Positive QFR (*n* = 48, 20.1%)	Total cohort (*N* = 239)	*p*-value
Age (years), median [IQR]	84 [80–86]	84 [81–86]	84 [81–86]	*ns*
BMI (kg/m^2^), median [IQR]	26.2 [23.4–28.9]	24.8 [23.6–27.2]	26.0 [23.4–28.6]	*ns*
BSA (m^2^), median [IQR]	1.79 [1.66–1.92]	1.75 [1.67–1.87]	1.79 [1.66–1.90]	*ns*
Female, *n* (%)	111/191 (58.1)	26/48 (54.2)	137/239 (57.3)	*ns*
Hypertension, *n* (%)	174/191 (91.1)	44/48 (91.7)	218/239 (91.2)	*ns*
Diabetes, *n* (%)	52/191 (27.2)	15/48 (31.3)	67/239 (28.0)	*ns*
Dyslipidaemia, *n* (%)	120/191 (62.8)	30/48 (62.5)	150/239 (62.8)	*ns*
Smoking (current or former), *n* (%)	55/188 (29.3)	10/47 (21.3)	65/235 (27.7)	*ns*
History of CAD, *n* (%)	33/190 (17.4)	17/47 (36.2)	50/237 (21.1)	0.005
Previous AMI, *n* (%)	14/191 (7.3)	11/48 (22.9)	25/239 (10.5)	0.002
Previous PCI, *n* (%)	26/191 (13.6)	13/48 (27.1)	39/239 (16.3)	0.02
NYHA class, median [IQR]	3 [2–3]	3 [2–3]	3 [2–3]	*ns*
NYHA class I	5/182 (2.7)	1/46 (2.2)	6/228 (2.6)	*ns*
NYHA class II	38/182 (20.9)	14/46 (30.4)	52/228 (22.8)	*ns*
NYHA class III	119/182 (65.4)	22/46 (47.8)	141/228 (61.8)	*ns*
NYHA class IV	20/182 (11.0)	9/46 (19.6)	29/228 (12.7)	*ns*
CKD Stage I	16/183 (8.7)	5/45 (11.1)	21/228 (9.2)	*ns*
CKD Stage II	64/183 (35.0)	11/45 (24.4)	75/228 (32.9)	*ns*
CKD Stage III	89/183 (48.6)	25/45 (55.6)	114/228 (50.0)	*ns*
CKD Stage IV	12/183 (6.6)	4/45 (8.9)	16/228 (7.0)	*ns*
CKD Stage V	2/183 (1.1)	0 (0)	2/228 (0.9)	*ns*
COPD, *n* (%)	40/191 (20.9)	11/48 (22.9)	51/239 (21.3)	*ns*
Previous stroke/TIA, *n* (%)	24/191 (12.6)	6/48 (12.5)	30/239 (12.6)	*ns*
Active malignancy, *n* (%)	14/188 (7.4)	4/48 (8.3)	18/236 (7.6)	*ns*
Pre-existent LBBB, *n* (%)	14/191 (7.3)	3/48 (6.3)	17/239 (7.1)	*ns*
Pre-existent RBBB	10/191 (5.2)	2/48 (4.2)	12/239 (5.0)	*ns*
Pre-existent AF, *n* (%)	66/190 (34.7)	10/48 (20.8)	76/238 (31.9)	*ns*
Pacemaker, *n* (%)	12/191 (6.3)	3/48 (6.3)	15/239 (6.3)	*ns*
Previous balloon valvuloplasty, *n* (%)	13/191 (6.8)	4/48 (8.3)	17/239 (7.1)	*ns*
Previous valvular surgery, (%)				*ns*
Aortic valve surgery	4/191 (2.1)	0 (0)	4/239 (1.7)	
Mitral valve surgery	7/191 (3.7)	0 (0)	7/239 (2.9)	

AF, atrial fibrillation; AMI, acute myocardial infarction; BMI, body mass index; BSA, body surface area; CAD, coronary artery disease; CKD, chronic kidney disease; COPD, chronic obstructive pulmonary disease; IQR, interquartile range; LBBB, left bundle branch block; NYHA, New York Heart Association; PCI, percoutaneous coronary intervention; QFR, quantitative flow ratio; RBBB, right bundle branch block; TIA, transient ischemic attack.

Both QFR+ and QFR- groups were homogeneous for age, sex, and BMI (Table 1). No significant differences were found in terms of cardiovascular risk factors (i.e., arterial hypertension, diabetes mellitus, dyslipidemia, renal function, or smoking habit, Table 1). No significant differences in symptomatic status expressed by NYHA class were found between the two groups, the majority experienced a NYHA class III before TAVI procedure (Table 1). Valvuloplasty was used in a small percentage of the patients (17/239, 7.1%) and, in selected patients, as a mere bridge to TAVI, with no differences between the two populations and no following complications.

Overall, 50/237 (21.1%) patients had a history of CAD, with a significantly higher prevalence among patients with QFR+ (36.2% vs. 17.4%, *p* = 0.005, Table 1). Based on ECG parameters, no significant differences were detected between the groups in respect to intraventricular conduction delays or atrial fibrillation (Table 1). No significant differences were found in terms of other morbidities between the two groups, such as active malignancies, chronic obstructive pulmonary disease (COPD) or chronic kidney disease.

Echocardiographic data are shown in Supplementary Table S1. The two groups were homogeneous regarding aortic valve area (AVA) value, mean gradient and left ventricular ejection fraction (LVEF).

The analysis of the risk score (STS risk score) was similar between the two populations, and the procedural details of the TAVI regarding the type and size of the bioprosthetic do not have statistically significant differences (Supplementary Table S2).

### Overall data on coronary segments analysis

Descriptive data about the specific coronary segments are detailed in Table 2.

**Table 2:** Descriptive data of coronary segments analyzed by QFR.

	Negative QFR (*n* = 191, 79.9%)	Positive QFR (*n* = 48, 20.1%)	Total cohort (*N* = 239)
Left antererior discendent artery, LAD			
LAD RVD (mm), mean (SD)	2.71 (0.54)	2.62 (0.49)	2.70 (0.52)
LAD MLD (mm), mean (SD)	1.94 (0.57)	1.54 (0.50)	1.88 (0.58)
LAD area stenosis (%), mean (SD)	32.52 (14.01)	47.46 (14.22)	35.14 (15.25)^*^
LAD lesion lenght (mm), mean (SD)	15.21 (9.05)	24.44 (12.82)	17.00 (10.31)
LAD QFR, mean (SD)	0.94 (0.05)	0.78 (0.09)	0.90 (0.08)
Left circumflex artery, LCx			
LCx RVD (mm), mean (SD)	2.76 (0.60)	2.77 (0.59)	2.76 (0.58)
LCx MLD (mm), mean (SD)	2.11 (0.87)	1.89 (0.68)	2.06 (0.81)
LCx area stenosis (%), mean (SD)	26.28 (15.27)	36.60 (20.96)	28.28 (16.86)^*^
LCx lesion lenght (mm), mean (SD)	13.05 (8.89)	14.29 (7.87)	13.19 (8.44)
LCx QFR, mean (SD)	0.97 (0.03)	0.93 (0.08)	0.96 (0.05)
Right coronary artery, RCA			
RCA RVD (mm), mean (SD)	3.03 (0.76)	2.86 (0.48)	3.00 (0.70)
RCA MLD (mm), mean (SD)	2.34 (0.77)	1.79 (0.74)	2.23 (0.78)
RCA area stenosis (%), mean (SD)	26.89 (18.77)	41.19 (24.85)	29.56 (20.7)^*^
RCA lesion lenght (mm), mean (SD)	12.95 (9.16)	15.45 (9.19)	13.51 (8.96)
RCA QFR, mean (SD)	0.96 (0.04)	0.87 (0.12)	0.94 (0.07)

LAD, left anterior descendant artery; LCx, left circumflex artery; MLD, minimum lumen diameter; QFR, quantitative flow ratio; RCA, right coronary artery; RVD, reference vessel diameter; SD, standard deviation.

^*^ Patients in the QFR+ group had a significantly higher mean percent stenosis across all vessels (QFR+ vs. QFR-: for LAD *p* < 0.001; for LCx *p* = 0.002; and for RCA *p* < 0.001).

Overall, mean of Minimum Lumen Diameter (MLD) was lower for LAD vs. the other two branches, with a higher percentage of area stenosis (LAD 35.14% vs. LCx 28.2% vs. RCA 29.56%).

Patients in the QFR+ group had a significantly higher mean percent stenosis across all vessels (QFR+ vs. QFR-: for LAD *p* < 0.001; for LCx *p* = 0.002; and for RCA *p* < 0.001). Therefore, no vessel analyzed had a coronary artery stenosis mean rate greater than 50%, and according to the latest consensus document, none of the patients underwent PCI (36). QFR value among the three branches (LAD, LCx and RCA) was lower for LAD, most frequently involved in QFR+ population (37/48, 77,1%), as shown in Supplementary Table S3.

### Long-term outcomes according to QFR value

**Figure 2:**
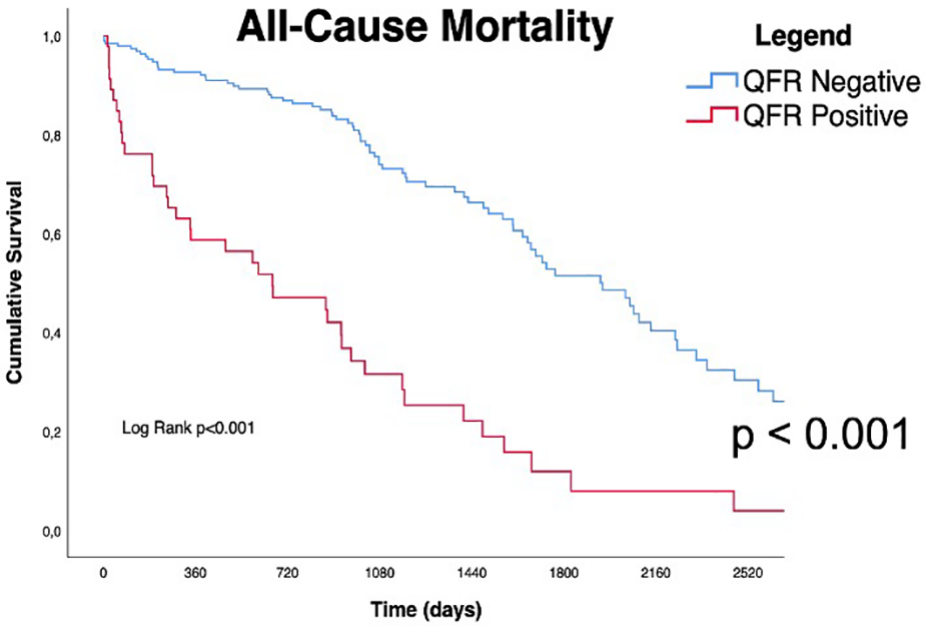
Kaplan Meyers curves for all cause of mortality. Red line represents QFR+ cohort trend during the follow up period, blue line represents QFR- cohort during the follow up period. the median follow-up was 1,005 days [IQR 582-2218]. QFR, quantitative flow ratio.

Long-term outcomes according to QFR are shown in Table 3, the median follow-up was 1,005 days [IQR 582-2218]. About half of all patients died during follow-up (122/239, 51%). As shown in Table 3 and by the Kaplan-Meier analysis (Central Illustration and Figure 2), patients who had a positive QFR value on any of the analyzed branches had a significantly higher all-cause mortality (81.3% vs. 43.5%, *p* < 0.001).

**Table 3:** Long-term outcomes according to QFR: the median follow-up was 1,005 days [IQR 582-2218].

	Negative QFR (*n* = 191, 79.9%)	Positive QFR (*n* = 48, 20.1%)	Total cohort (*N* = 239)	*p*-value
All-cause mortality, *n* (%)	83/191 (43.5)	39/48 (81.3)	122/239 (51.0)	<0.001
Composite outcome^a^, *n* (%)	113/175 (64.6)	39/44 (88.6)	152/219 (69.4)	0.002
Cardiovascular mortality, *n* (%)	44/190 (23.2)	28/48 (58.3)	72/238 (30.3)	<0.001
Stroke/TIA, *n* (%)	23/162 (14.2)	3/39 (7.7)	26/201 (12.9)	0.27
Myocardial infarction, *n* (%)	7/162 (4.3)	1/39 (2.6)	8/201 (4.0)	0.61
Major bleedings, *n* (%)	17/164 (10.4)	5/38 (13.2)	22/202 (10.9)	0.62
Any hospitalization, (%)	47/157 (29.9)	11/36 (30.6)	58/193 (30.1)	0.94

QFR, quantitative flow ratio; TIA, transient ischemic attack.

^a^ Includes all-cause mortality, cardiovascular mortality, stroke/TIA, myocardial infarction, major bleedings, any hospitalization.

**Figure 3:**
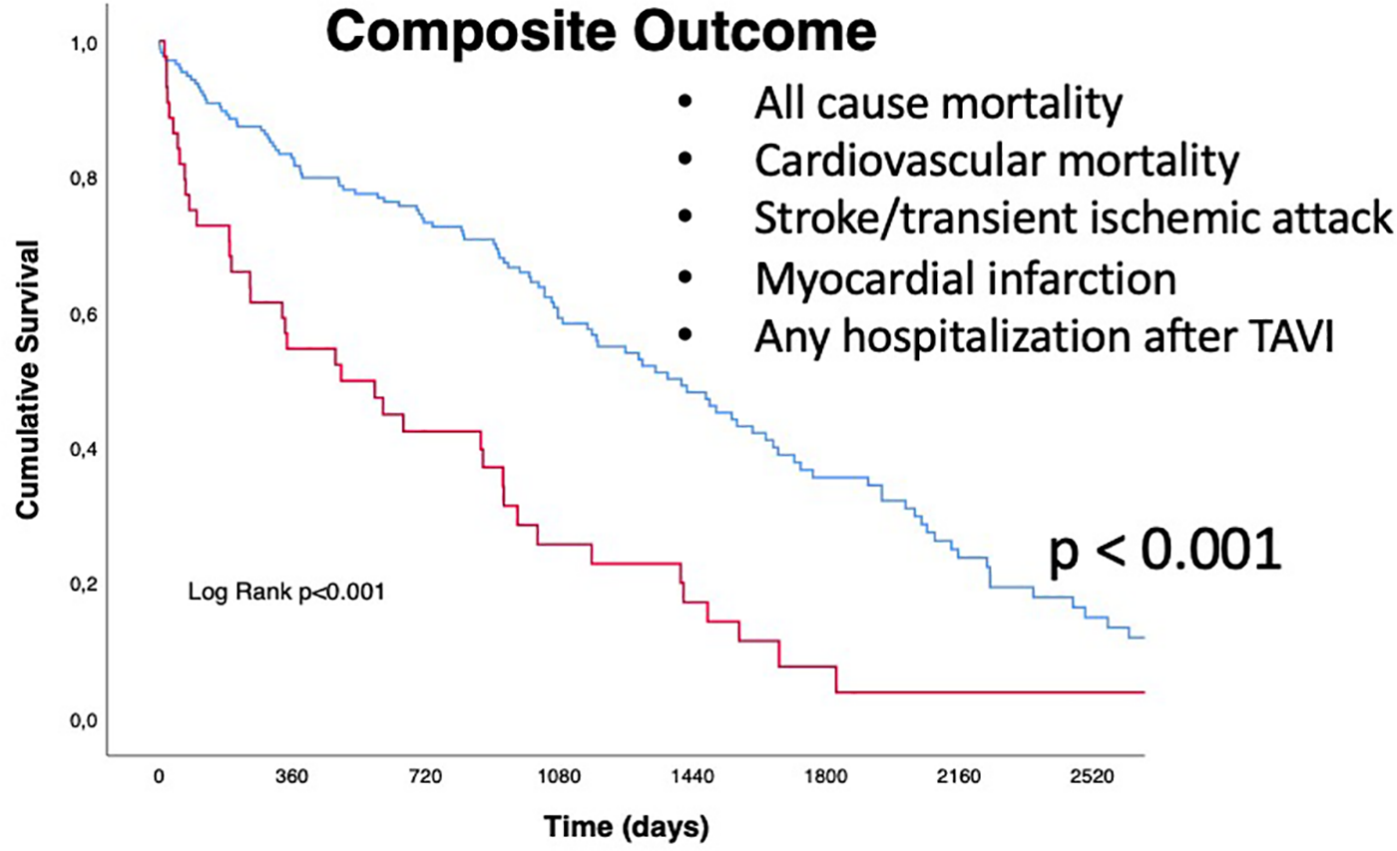
Kaplan Meyers curves for secondary outcome (composite of all-cause mortality, cardiovascular mortality, stroke/transient ischemic attack (TIA), myocardial infarction (MI), and any hospitalization after TAVI). Red line represents QFR+ cohort trend during the follow up period, blue line represents QFR- cohort during the follow up period. The median follow-up was 1,005 days [IQR 582-2218]. QFR, quantitative flow ratio; TAVI, transcatheter aortic valve implantation, TIA, transient ischemic attack.

Regarding the secondary endpoint (the composite of all cause of death, cardiovascular mortality rate, acute cerebrovascular events such as stroke or TIA, acute myocardial infarction, and any hospitalization following TAVI), patients in the QFR+ group had a significantly higher rate of events (88.6% vs. 64.6%, *p* = 0.002) (Table 3, Central Illustration and Figure 3) In more detail, cardiovascular mortality rate was the main difference between the two populations (QFR+ 58.3% vs. QFR− 23.2%, *p* < 0.001). For the other outcomes of interest, there was no significant differences among the two groups (Table 3).

On the adjusted Cox regression analysis, patients with positive global QFR were independently associated with a higher risk of all-cause mortality (Model 1, HR 3.47, 95% CI, 2.35−5.12; Model 2, HR 5.01, 95% CI, 3.17−7.90) compared to patients with a negative QFR. Similar results were found for the composite outcome (Table 4).

**Table 4:** Unadjusted and adjusted Cox regression analyses.

	Unadjusted		Model 1		Model 2	
	HR [95% CI]	*p*	aHR [95% CI]	*p*	aHR [95% CI]	*p*
All-cause mortality						
QFR negative (ref)	ref		ref		ref	
QFR + not involving LAD	1.86 [0.86–4.05]	0.11	1.99 [0.91–4.34]	0.08	3.21 [1.31–8.43]	0.01
QFR + involving LAD	4.45 [2.92–6.79]	<0.001	4.22 [2.75–6.46]	<0.001	5.61 [3.41–9.21]	<0.001
Composite outcome^a^						
QFR negative (ref)	ref		ref		ref	
QFR + not involving LAD	1.62 [0.75–3.49]	0.21	1.65 [0.76–3.56]	0.20	1.54 [0.62–3.82]	0.34
QFR + involving LAD	2.60 [1.73–3.89]	<0.001	2.44 [1.62–3.68]	<0.001	3.19 [2.01–5.06]	<0.001

LAD, left anterior descendant artery; LCx, left circumflex artery; MLD, minimum lumen diameter; QFR, quantitative flow ratio; RCA, right coronary artery; RVD, reference vessel diameter; SD, standard deviation; aHR, adjusted hazard ratio.

Model 1 was adjusted for age and sex.

Model 2 was adjusted for age, sex, hypertension, diabetes, coronary artery disease, CKD, previous stroke, malignancy and atrial fibrillation.

^a^ Includes all-cause mortality, cardiovascular mortality, stroke/TIA, myocardial infarction, major bleedings, any hospitalization; composite outcome of all-cause mortality/myocardial infarction/stroke/TIA/any hospitalization.

Furthermore, in the adjusted Cox regression analysis, patients with QFR+ involving LAD were associated with a significant higher risk of all-cause mortality and the composite outcome compared to patients with negative QFR or QFR + not involving LAD (Table 4).

### Comparison between angiographic evaluation of coronary lesion and QFR assessment

Among the 239 patients, a total of 23 (9.6%) patients had at least one coronary lesion of ≥70% diameter stenosis, while 216/239 (90.4%) had no significant CAD.

In detail, 4 patients had a stenosis ≥70% involving LAD (2 proximal LAD, 1 apical LAD and 1 mid LAD); 6 patients had a stenosis ≥70% involving LCx (3 intermediate LCx and 3 proximal LCX); 12 patients had a stenosis ≥70% involving RCA (7 proximal RCA; 2 distal RCA and 2 mid RCA); 1 patient had a stenosis ≥70% involving both RCA and LCx (intermediate LCx and mid RCA).

As expected, patients with QFR+ had a significantly higher prevalence of severe CAD (at least one coronary artery lesion ≥70% diameter stenosis) compared to patients with negative QFR (14/48 [29.2%] vs. 9/191 [4.7%], *p* < 0.001). However, as shown in Supplementary Table S4, crude rates of all-cause mortality and the composite outcomes were similar among the two groups (i.e., lesion stenosis ≥70% vs. <70%) without statistically significant differences (52.2% vs. 50.9%, *p* = 0.91 for all-cause mortality; 77.8% vs. 68.7%, *p* = 0.42 for the composite outcome). These results were confirmed also at the Cox regression analysis for both outcomes even after the adjustments for age and sex (aHR 1.62, 95% CI, 0.88−2.97, *p* = 0.11 for all-cause mortality and aHR 1.55, 95% CI, 0.88−2.69, *p* = 0.12).

## Discussion

This study emphasizes the importance of the functional assessment of intermediate coronary artery stenosis and proposes a non-invasive systematic method such as QFR in the workflow management of patients with severe AS considered for TAVI. QFR performance accuracy is already well defined in chronic CAD and non-culprit lesions of patients with acute coronary syndromes (37–39).

In addition, the effect of adenosine used in the hyperemia-based coronary function study may be attenuated in patients with AS, leading to a potential underestimation of the severity of coronary stenosis with FFR.

The most recent Guidelines recommend considering PCI in patients with a primary indication to undergo TAVI and with coronary artery diameter stenosis >70% in proximal segments proximal segments. However, the Recommendation Class is IIa with a weak “C” Level of Evidence (7).

The recent Consensus Document emphasizes that PCI should be considered in patients with severe AS with primary indication for TAVI who reveal 70% or more DS lesions in proximal coronary segments, thus affecting a large area of myocardium at risk, or in patients with angina (18, 34).

Lateef et al. with a 5,000 patients meta-analysis showed that were no benefits coming from PCI in TAVI patients for 30 days MACE and one-year mortality (14). Results from the Assessing the Effects of Stenting in Significant Coronary Artery Disease Prior to Transcatheter Aortic Valve Implantation (ACTIVATION) trial recently demonstrated that rates of mortality and rehospitalization at 1 year were similar between PCI and no PCI prior to TAVI, non-inferiority margin was even not met and actually PCI was not without risks, resulting in a higher incidence of bleeding at 1 year (40). Recent REVASC-TAVI registry add an important piece to this puzzle, asserting that in patients undergoing TAVI, the completeness vs. incompleteness of myocardial revascularization is an equivalent strategy in reducing the rate of all cause death, as well as the risk of stroke, myocardial infarction, and rehospitalization for heart failure at 2 years, regardless of the clinical and anatomical situations (41).

Surprisingly, one of the most striking observations that emerges from our observational analysis was that QFR+ population has worse outcomes and thus need more in-depth evaluation on a patient-by-patient basis. The strength of this study is the QFR-based stratification of epicardial stenosis in patients in whom the efficacy of combining PCI and TAVI is often questionable. This new functional index sheds new light on this debated topic and unlike the simple angiographic criteria that are inherently dependent on operators, this assessment allows a prognostic evaluation supported by previous evidence.

It should also be specified that although the risk of unplanned PCI after TAVI is low in patients who do not have CAD at the time of TAVI, it accumulates over time in patients who have subcritical CAD, especially if multiple lesions occur on multiple vessels (42).

Our data, even if limited, suggest that functional assessment in these cases can provide a greater and more specific accuracy, and that it could be applied on a large scale by integrating assessment criteria at the Heart Team. The QFR data has an important meaning in view of the prognostic significance of increasingly younger patients, as in the case of TAVI. Our data suggest that functional assessment in these cases can provide a greater and more specific accuracy, and that it could be applied on a large scale by integrating assessment criteria at the Heart Team. The QFR data has important significance in view of the prognostic significance of increasingly younger patients, as in the case of TAVI. Even in surgical risk scores, the QFR figure could bring benefits and determine the Heart Team's final choice. Therefore, tailoring of this approach allows to identify significant CAD and permits to detect patients at high risk of mortality or MACE. QFR analysis is a functional adenosine-free and off-line investigation: we hypothesize that, if routinely performed and considered by local Heart Teams, would allow a reduction in residual cardiovascular risk beyond valve disease.

Statistical significance focuses on functionally positive lesions for LAD. Patients with a QFR >0.80 on LAD were the majority of the QFR+ cohort (37/48, 77.1%), and when isolated from the remaining patients with positive QFR (i.e., not involving LAD 2.9%), they have worse outcomes. It is known that the myocardial mass served by LAD is greater than that managed by the other two vessels, the left circumflex branch and the right coronary artery, so the proportion of functionally ischemic myocardium is greater in these patients (43), driving hard endpoints.

Furthermore, our data suggest that a functional assessment by QFR could be more meaningful in predicting long-term events than an assessment by the mere angiographic stenosis rate, which was not significantly associated with the long-term outcomes considered.

PCI based on the QFR derived from pre-TAVI ICA, if indicated, could be easily performed before, meanwhile or after the procedure on aortic valve.

Furthermore, coronary re-access after TAVR is often challenging and represents a great concern with important implications for the management of patients with severe AS. Previous studies have described in detail the challenge of selective coronary access for PCI after TAVR, especially in the presence of supra-annular devices with high stent frames (44–46). In RE-ACCESS, unsuccessful coronary cannulation was observed in 7.7% of patients with a previously implanted THV, and semi-selective cannulation was not rare (12.0%, for the left coronary artery and in 31.7% for the right coronary artery) (45).

This finding has important clinical and procedural implications for the management of the patients with severe AS. The different anatomical characteristics that lead to the choice of a specific type of THV deeply influence future re-access to coronary ostia, as well as new implantation projections (seeking the best commissural alignment), mainly related with self-expandable systems.

As a final alternative, if the patient's condition is good after THV release in TAVI procedure, by selectively cannulating coronary ostia, QFR analysis could allow immediate and objective assessment of the severity of CAD, with the dual purpose of assessing both the easiness of the re-access to the coronary ostia and the functional features of the epicardial stenosis.

Likewise, QFR could reduce the need for subsequent, sometimes unnecessary, assessment of intermediate CAD by a new ICA or by stress testing, thereby reducing patient risk and healthcare costs. Finally, the threadless nature of the QFR would limit the potential difficulties of entering the coronary ostia with a pressure wire through the TAVI valve meshwork.

## Limitations

This study has some limitations: firstly, it retrospectively considers data from all patients undergoing TAVI, some of whom had a history of coronary angioplasty, so this, while not altering the QFR data, unbalances the population with prior PCI in the QFR+ arm. For the “prior PCI” data, the authors considered any type of balloon or stent angioplasty on any coronary segment prior to TAVI.

Another important limitation of this study is related to its retrospective observational design. Thus, angiographic description and quantification of CAD is missing, and Syntax Score was not systematically collected in all cases, there is no core laboratory analysis of procedural results or independent adjudication of clinical events, with data drawn from four large centers with different filing systems. Nevertheless, functional assessment with QFR has been shown to be superior to mere angiographic data (35, 47).

Our study population is not a large cohort, anyway it explores a data set from the real-world population and proposes hypotheses that can be tested in future studies.

## Conclusions

Pre-TAVI QFR could be used for a safe, quick, and simple, wireless functional assessment of CAD before TAVI procedures. This would allow the physicians to assess the potential indication for a myocardial revascularization procedure based on QFR values. If indicated, this would allow PCI to be performed before TAVI or anyway schedule an appropriate surveillance for planning a PCI with the aim to positively influence the adverse events occurring in subsequent years of follow-up that our study outlined in patients with positive QFR values at baseline coronary angiogram. Undoubtedly, more data are needed to assess the clinical value of functional coronary assessment in patients with severe AS. This study raises the need for studies focused on functionally significant coronary arteries stenoses in patients with severe AS undergoing TAVI. Ongoing research such as TransCatheter Valve Vessels Trial (ClinicalTrials.gov: NCT03424941) and COMPLETE TAVR (ClinicalTrials.gov: NCT04634240) will allow to appropriately assess the real value of incorporating physiology-guided PCI in TAVI patients and to clarify the most correct approach to CAD in this population.

## Data Availability

The raw data supporting the conclusions of this article will be made available by the authors, without undue reservation.
