# Supplementary material for: Quantitative flow ratio-based outcomes in patients undergoing transcatheter aortic valve implantation quaestio study
Source: Front Cardiovasc Med. 2023 Aug 30;10:1188644. doi: 10.3389/fcvm.2023.1188644 (PMC10499393; doi:10.3389/fcvm.2023.1188644)
Supplement: Supplementary file 1 [file Table1.docx]

**SUPPLEMENTARY MATERIALS**

**Supplementary Table 1.** Echocardiographic data of patients analysed.

| **Parameters** | **Negative QFR** (n=191, 79.9%) | **Positive QRF**  (n=48, 20.1%) | **Total cohort**  (N=239) | *p value* |
| --- | --- | --- | --- | --- |
| Trans-aortic mean gradient (mmHg), median [IQR] | 46 [40-58] | 45.5 [41.2-53.2] | 46 [40-56] | *ns* |
| AVA (cm2), median [IQR] | 0.7 [0.6-0.8] | 0.8 [0.65-0.90] | 0.7 [0.6-0.9] | *ns* |
| **Left ventricular function** |  |  |  |  |
| Preserved (LVEF>50%) | 132/181 (72.9) | 33/46 (71.7) | 165/227 (72.7) | *ns* |
| LVEF 40% to 50% | 28/181 (15.5) | 6/46 (13.0) | 34/227 (15.0) | *ns* |
| LVEF 30% to 39% | 14/181 (7.7) | 6/46 (13.0) | 20/227 (8.8) | *ns* |
| LVEF <30% | 7/181 (3.9) | 1/46 (2.2) | 8/227 (3.5) | *ns* |

**Legend:** AVA, Aortic Valve Area; LVEF, Left Ventricular Ejection Fraction; IQR, Interquartile Range; QFR, Quantitive Flow Ratio;

**Supplementary Table 2. Patients’ STS risk scores and TAVI procedural specifics: type and size of THV implanted.**

|  | **Negative QFR** (n=191, 79.9%) | **Positive QRF**  (n=48, 20.1%) | **Total cohort**  (N=239) | *p value* |
| --- | --- | --- | --- | --- |
| **STS risk score category, n (%)** |  |  |  |  |
| Low (STS score < 4%) | 120/184 (65.2) | 24/47 (51.1) | 144/231 (62.3) | *ns* |
| Intermediate (4%≤ STS score <8%) | 55/184 (29.9) | 22/47 (46.8) | 77/231 (33.3) | *ns* |
| High (STS score >8%) | 9/184 (4.9) | 1/47 (2.1) | 10/231 (4.3) | *ns* |
| **Procedural specifics** |  |  |  |  |
| **Type of valve,** n (%) |  |  |  |  |
| Self-expandable | 110/188 (58.5) | 30/46 (65.2) | 140/234 (59.8) | *ns* |
| Balloon expandable | 78/188 (41.5) | 16/46 (34.8) | 94/234 (40.2) | *ns* |
| **Prosthetic valve size** (mm)**,** median [IQR] | 26 [23-29] | 26 [23-29] | 26 [23-29] | *ns* |

**Legend:** STS, Society of Thoracic Surgeons; QFR, Quantitative Flow Ratio.

**Supplementary Table 3**. Long term outcomes according to QFR involving or not involving LAD, median follow-up of 1005 days [IQR 582-2218].

|  | **Negative QFR** (n=191, 79.9%) | **Positive QRF not involving LAD**  (n=11, 4.6%) | **Positive QRF involving LAD**  (n=37, 15.5%) | *p value* |
| --- | --- | --- | --- | --- |
| **Composite outcome^†^,** n (%) | 122/194 (62.9) | 7/10 (70.0) | 35/38 (92.1) | <0.001 |
| **All-cause mortality,** n (%) | 113/175 (64.6) | 7/9 (77.8) | 32/35 (91.4) | 0.006 |

Legend: LAD, Left Anterior Descendant artery; QFR, Quantitative Flow Ratio.

**Supplementary Table 4**. Long-term outcomes according to patients with severe CAD (i.e. patients with at least one coronary artery lesion ≥70%) vs no significant CAD.

|  | **Coronary artery**  **lesion <70%**  (n = 216, 90.4%) | **At least one coronary artery lesion ≥70%** (n = 23, 9.6%) | *p value* |
| --- | --- | --- | --- |
| **Composite outcome^†^,** n (%) | 138/201 (68.7%) | 14/18 (77.8%) | 0.42 |
| **All-cause mortality,** n (%) | 110/216 (50.9) | 12/23 (52.2) | 0.91 |

**^†^**Composite outcome of all-cause mortality / CV-death / myocardial infarction / stroke / TIA / any hospitalization

**Supplementary Figure 1.** Workflow chart of Contrast Vessel QFR analysis.


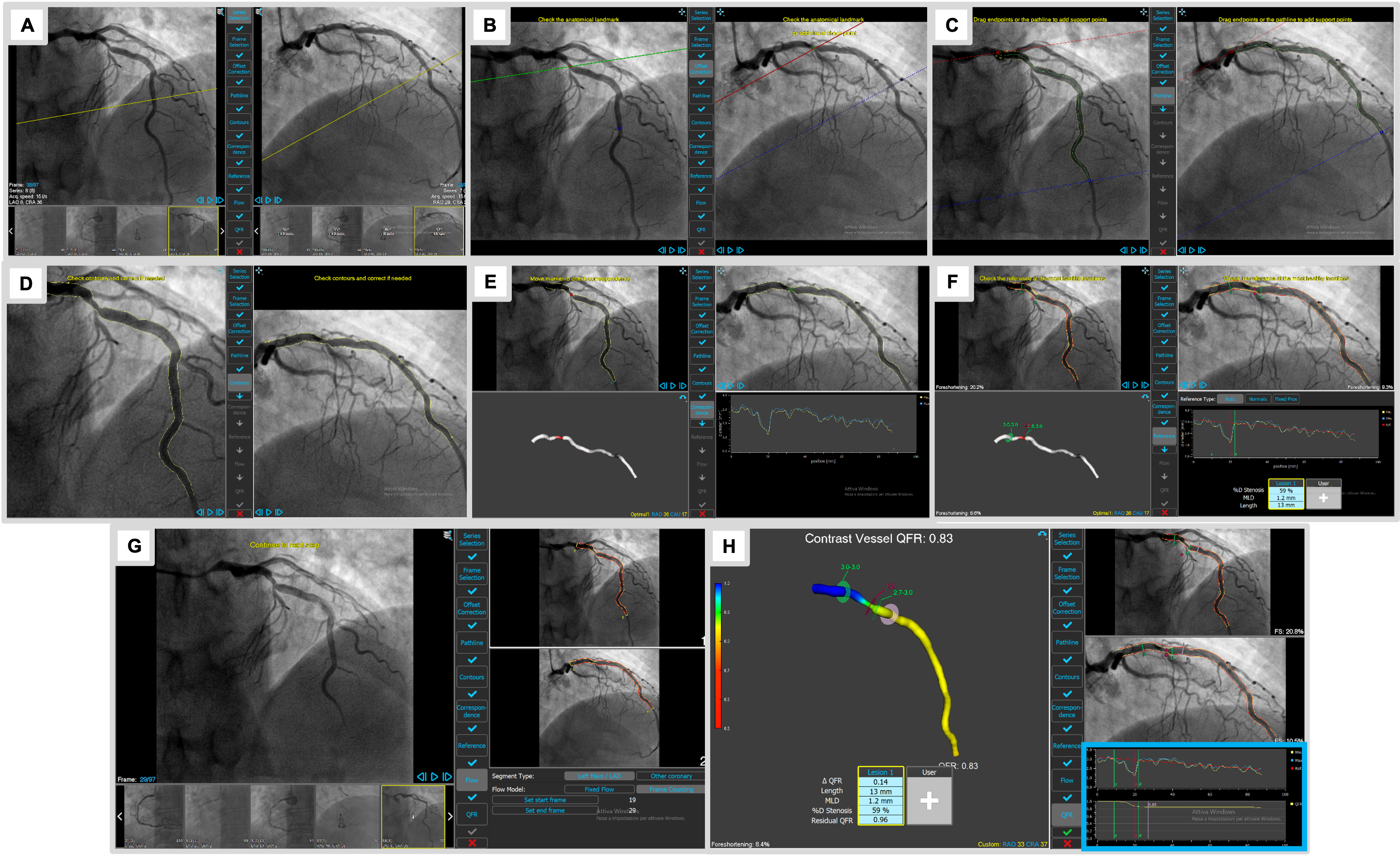


1. Selection of two orthogonal series (at least 25-degrees) with good representation of the target vessel, avoiding overlap with other vessels, tele-diastole frames selection with complete contrast filling, after intracoronary 100-200 mcg nitroglycerin injection confirmation;
2. Offset correction: selection of a coronary landmark (e.g., a bifurcation), coincident on both series selected;
3. Define the starting point (ideally the ostium) and ending point of vessel analysis. Possible manual path line correction;
4. Evaluate contours automatically: the software can recognize the contrast delineated contours of the vessel, but the operator can eventually manually correct them;
5. Assess the accuracy of the 3D-reconstruction of the vessel, looking for the match of the 2 lines (see low right blue quadrant);
6. Automatic calculation of intermediate stenosis degree and selection of reference stenosis;
7. Frame counting selection of the starting frame and of the ending frame, corresponding to the first frame in which the medium contrast reaches the proximal point of analysis in the vessel and the frame in which it reaches the distal point, respectively;
8. Final and complete analysis summary, with the Contrast Vessel QFR value obtained;

Main exclusion criteria for a good QFR analysis are: low coronary angiography images quality, absence of valid projections, excessive vessel overlaps or tortuosity, coronary aneurysms, aorto-ostial lesions;
